# A Review on Recent Deep Learning-Based Semantic Segmentation for Urban Greenness Measurement

**DOI:** 10.3390/s24072245

**Published:** 2024-03-31

**Authors:** Doo Hong Lee, Hye Yeon Park, Joonwhoan Lee

**Affiliations:** 1 Landscape Architecture and Environmental Planning, College of Agriculture and Applied Sciences, Utah State University, Logan, UT 84322, USA; doohong.lee@usu.edu; 2 School of Planning, College of Design, Architecture, Art, and Planning, University of Cincinnati, Cincinnati, OH 45221, USA; park2ha@ucmail.uc.edu; 3 Division of Computer Science and Engineering, Jeonbuk National University, Jeonju 54896, Republic of Korea

**Keywords:** urban green space (UGS), greenness measures, deep learning (DL)-based semantic segmentation, landscape analysis and planning

## Abstract

Accurate urban green space (UGS) measurement has become crucial for landscape analysis. This paper reviews the recent technological breakthroughs in deep learning (DL)-based semantic segmentation, emphasizing efficient landscape analysis, and integrating greenness measurements. It explores quantitative greenness measures applied through semantic segmentation, categorized into the plan view- and the perspective view-based methods, like the Land Class Classification (LCC) with green objects and the Green View Index (GVI) based on street photographs. This review navigates from traditional to modern DL-based semantic segmentation models, illuminating the evolution of the urban greenness measures and segmentation tasks for advanced landscape analysis. It also presents the typical performance metrics and explores public datasets for constructing these measures. The results show that accurate (semantic) segmentation is inevitable not only for fine-grained greenness measures but also for the qualitative evaluation of landscape analyses for planning amidst the incomplete explainability of the DL model. Also, the unsupervised domain adaptation (UDA) in aerial images is addressed to overcome the scale changes and lack of labeled data for fine-grained greenness measures. This review contributes to helping researchers understand the recent breakthroughs in DL-based segmentation technology for challenging topics in UGS research.

## 1. Introduction

The global demographic is undergoing a significant shift, with approximately 57% of the world’s population residing in urban areas as of 2022, with a notable anticipated increase of 66% by the year 2050 [1]. As urban populations continue to grow, urban areas become more densely populated, cities expand in size, and the need for urban residents to access nature either physically or visually becomes more necessary. The reduction in green spaces serve as a pivotal point for analyzing both their quantity and quality. According to health studies, this reduction is associated with occurrences of physical or mental health issues [2,3]. Additionally, from a sociological perspective, the reduction exacerbates inequalities in human well-being, adversely affecting residents [4,5].

Urban green spaces (UGSs) are defined in land-use planning as open areas reserved for parks and other natural environments, including street trees, flora, water features, and manicured lawns [6,7]. Therefore, due to their provision of services, UGSs are considered a crucial component in urban planning, subject to precise measurement and management.

There are multiple ways of classifying UGS measures, such as exposure types in terms of geographical aspects [7] or the perceived nature in terms of psychological aspects [8]. However, few studies have considered how the data generated or measured from viewing greenness can be used to classify these measures. One study discusses how different pixel values were detected based on the view direction of the image data [9]. By adopting this idea, we suggest a novel approach based on the view directions of image processing to classify the measures. What stands out in this approach is the classification of UGS measurements into those based on the plan view (top-view or ortho-mapped photos) and those based on the perspective view, depending on the viewing direction and visualization method. Although they are not explicitly stated, this review includes various potential approaches to improve the current UGS measures with the help of recent technological breakthroughs. The research purpose of this paper is to narrow the scope of the topics and provide a more in-depth introduction to the latest deep learning-based semantic segmentation models for accurate and fine-grained greenness measurements from perspective and aerial (satellite) image analysis.

**Figure 1:**
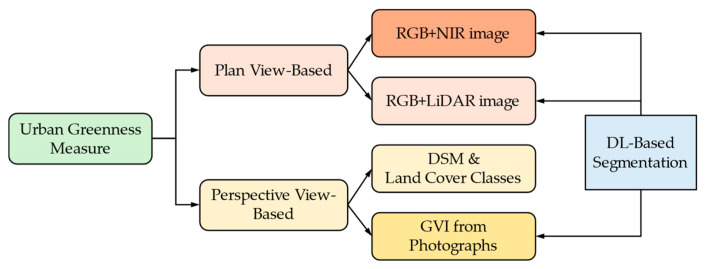
Urban greenness measures and how to calculate them with DL-based segmentation.

Figure 1 shows the taxonomy of the urban greenness measures used in this paper, along with the corresponding calculation approaches. The plan view, which can be conceptualized as a view from the sky like that from a satellite or plane, is a greenness measure directly derived from the red, green, and blue (RGB) colors used on a digital display screen or from multispectral aerial images. This contrasts with the perspective view that we, on the ground, typically perceive [10,11]. The image with better spatial resolution can look more closely at the green objects in an area of interest, which implies that the finer categories of green objects can be discerned for measuring the more meaningful measures. In general, for the plan view measurements, the spatial resolution of the images is more important than the frequency resolution, as green objects can be well identified through the visible RGB and NIR (Near-Infrared) bands. The ultimate goal of the plan view image analysis is Land Cover Classification (LCC) from RGB or RGB+NIR passive images, although active images, such as LiDAR (Light Detection and Ranging) images, can be supplemented to obtain more accurate and fine-grained green spaces [11,12,13].

Conversely, the perspective view measures involve visibility analyses, such as viewshed or visual-magnitude (VM) analyses, using three-dimensional Digital Terrain Maps (DTMs) or Digital Surface Maps (DSMs) and the two-dimensional LCC results from plan view images [13,14]. DTMs and DSMs are raster-based images used as maps representing the heights of the pixels. By subtracting a DSM from a DTM, urban studies can determine the heights and locations of structures, such as buildings and trees, rising from the ground level. LiDAR or stereo-imaging techniques play a pivotal role in reconstructing DTMs and DSMs [15]. In addition, the greenness based on the perspective view can be measured using two-dimensional, perspective-mapped photographs. The Green View Index (GVI) uses Google Street View (GSV) photographs, taken by cars equipped with cameras on top, which alleviates the challenges of the data acquisition process [16]. Thus, accurate DSM and DTM construction with the plan view LCC is important to obtain the viewshed- or VM-based greenness, and the exact image analysis of 2D photographs is essential for the GVI [17]. This review paper focuses on the issues concerning greenness measurement based on the GVI.

This review delves into the recent technological progress in image segmentation based on deep learning (DL) techniques, which provide automatic analysis results as a component of urban visual intelligence [7]. One of the characteristics of DL technology is that it requires a huge amount of data instead of a human expert’s intervention, regardless of whether labeled or unlabeled data are used. Along with the typical metrics for semantic segmentation, we also introduce aerial-imagery and urban-street datasets that can be used for DL-based plan view greenness and GVI calculations, respectively. We try to focus on DL-based semantic segmentation for the greenness measures, but the recent segmentation model has evolved to deal with instances of an object or even parts of an instance that are potentially available for advanced landscape analysis. In general, there are no clear boundaries of the green or related objects in urban greenness measures. For instance, some studies may focus solely on tree cover to count the greenness measure, as in the GVI [16], while others consider various fine-grained green objects [18]. Thus, an efficient strategy involves constructing a dataset that includes green objects relevant to the study’s objectives. All these aspects are briefly and inclusively addressed in this review.

In the Discussion Section, we explore two critical aspects: (1) the use of semantic segmentation-based decision making for fine-grained greenness measures and qualitative landscape analysis with an emphasis on explainability, and (2) dataset construction and unsupervised domain adaptation. The drawback of black-box models for decision making lies in their lack of explainability. While DL-based decisions may be accurate, the incompleteness of the explainability poses issues, particularly in qualitative landscape analysis for planning. To address this, post-segmentation by DL-based models along with proper visual indicators can be combined with regression models for the quality evaluation of the landscape to emulate human evaluators, providing a more direct relationship between the segments and landscape quality evaluation. Additionally, employing unsupervised domain adaptation (UDA) is especially crucial for the plan view greenness measure to overcome the scale changes and lack of labeled aerial data using a large number of unlabeled data [18,19].

The contents of this review can be summarized as follows:

- The characterization and categorization of greenness measures into the plan view and the perspective view categories;
- The introduction of recent DL-based semantic segmentation models, constructed from Convolutional Neural Networks (CNN) and Visual Transformers, along with the corresponding datasets and performance evaluation measures;
- The proposal of a fine-grained greenness measure and the combined approach involving semantic segmentation and regression models for a more intuitive qualitative landscape evaluation, addressing the limitations of black-box decision making;
- The introduction of UDA as a solution to the labeled-data issue in semantic segmentation for calculating greenness measures based on the plan view.

**Figure 2:**
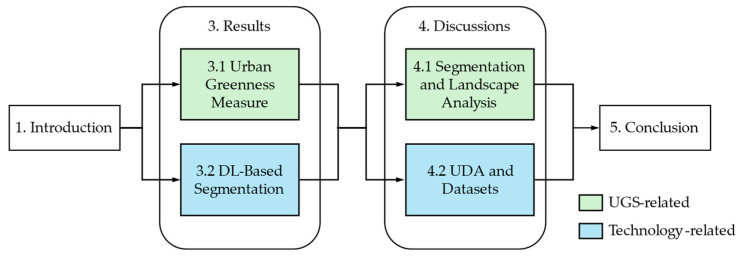
Section organization and related contents of this review.

Because the potential readers of this review are presumably landscape researchers and AI application engineers, we tried to arrange the sections to satisfy their different needs. For AI application engineers, Section 3.1 reviews the plan view and the perspective view greenness measures. Then, Section 3.2 introduces carefully chosen DL-based segmentation models with available datasets for landscape researchers. In Section 4, we discuss a couple of issues: (1) the inevitability of segmentation for the explainable quality analysis of the landscape for planning, and (2) the necessity of UDA for aerial images, with a discussion on the potential avenues for further research on the greenness measures based on the plan view. The former might be helpful to choose methods for advanced landscape analysis, while the latter is an important issue for AI-based remote sensing. Finally, Section 5 presents a summary of this comprehensive review. Figure 2 shows the section organization with the related contents.

## 2. Methods

This review provides a narrative explanation of topics within the designated scopes, which necessitated the qualitative selection of review articles. The methodology of this study emphasizes the necessity for a review that enhances the comprehension between research utilizing existing data and research generating and analyzing data. Consequently, we conducted a review spanning two distinct fields that can be interlinked. All references were sourced from English-language publications and underwent peer review or validation through conferences within their respective disciplines. All cited figures are included with the original authors’ permission, and the necessary rights to use the figures were acquired from the publishers, ensuring compliance with copyright regulations.

To enhance the understanding of this review, we first introduce a brief background on the urban greenness measures and how to calculate them, which requires the background on semantic segmentation. Then, we delve into the introduction of DL-based semantic segmentation techniques for greenness measurements and landscape analysis. This presentation includes an introduction to the relevant datasets and typical performance evaluation metrics.

## 3. Results

### 3.1. Fundamentals of Urban Greenness Measures

In this section, we provide a brief introduction to the foundational aspects of the greenness measures, covering their applications, the various forms of green-space exposures experienced, and the characterization methods employed. Additionally, the calculations of the plan view and the perspective view greenness measures and how the segmentation technique is exploited are discussed.

#### 3.1.1. Brief Background of Urban Greenness Measures

Before we address the specific measures in this review, we offer an overview of the essential backgrounds to ensure a comprehensive understanding in the following review.

1. Usages of greenness measure

It is important to accurately evaluate the greenness in UGSs on which the environmental planning is based [5]. Also, epidemiological studies in recent decades have utilized various greenness measures to explore the relationship between green spaces and population-level health statuses [20,21]. Moreover, extensive research has been conducted on the relationship between urban green space and human well-being [22,23]. However, the two research fields of planning and their relationships with human health and well-being share commonalities in their measuring methods [24]. These methods involve assessing the extent of the greenness on a predefined scale, using geographic locations and information from spatial data, and leveraging technological breakthroughs, such as advanced computing and accurate spatial information.

The spatial scales range from the microscale to the macroscale, including the body/personal scale, the neighborhood scale, and the city/district scale [7]. Defining the spatial scale is a priority in determining the design of health and urban planning studies. In the following review, the semantic segmentation is related to the spatial scales by means of captured images for measuring greenness. For example, aerial images may be gathered at the city/district scale, while GSV corresponds well to the body/personal scale.

Notably, the research on greenness should assume a scale related to geographical locations including 2D maps. Also, the integration of information from spatial data is efficiently facilitated through the open-source-gathered Geographical Information System (GIS) and, subsequently, technological breakthroughs, such as DeepLabV3, are applied in commercial applications, such as Esri’s ArcGIS Pro 3.2 [25];

2. Spatially explicit forms of greenness exposure

How people experience their natural surroundings is one of the fundamental questions in the health benefits of urban green spaces [26]. There are three types of green-space exposure: availability, accessibility, and visibility. The availability of green space implies the physical amount of green space that actually exists [27,28]. The accessibility to green space refers to the spatial proximity of the green space to locations of interest [29]. The visibility of the green space stands for the amount of greenness that can be seen visually from a particular location of interest. The availability and accessibility can be captured objectively and quantitatively, while the visibility that is associated with “the amount of USG seen from the residents’ perspective” may include the quality of the green space [30]. Note that it is important to consider the 3D coordinates and perspective views of residents for evaluating the UGS visibility because tall residential buildings have become increasingly inevitable with the rapid progress of urbanization [31];

3. Quantitative vs. qualitative measures

Quantitative measures are generally focused on measuring the amount of UGS provided [32], which can be captured objectively. On the contrary, qualitative measurements deal with the characteristics related to the performance of the UGS, incorporating ecosystem services, which provide benefits from the natural environment to both humans and wildlife [33]. Recently, however, the UGS quality has been increasingly recognized for its effect on health [34,35]. In general, the assessment of the UGS quality is not obvious, and no universally accepted measurement is available [36,37,38].

In this review, we mainly focus on the quantitative greenness measure because the result of segmentation is given as a quantity in an objective manner. However, the goal of image analysis by segmentation goes beyond identifying green objects, such as trees, to calculating the associated quantitative measures. One can focus on several types of surrounding objects related to the quality of the green space in an image and define a quality measure from the segmentation results [10]. For example, one can analyze the spatial arrangement of the landscape elements allocated with green spaces to define a qualitative measure in an area after segmentation [39]. Sometimes it is necessary to measure the shape of the green spaces in the plan view images, namely, the green-space morphology [40]. Thus, the segmentation should be accurate for diverse objects related to landscape analysis and planning. Later, in Section 4.1, the necessity of semantic segmentation will be discussed in detail.

#### 3.1.2. How to Calculate Urban Greenness Measures

As expressed in Figure 1, there are two types of greenness measures based on the plan view and the perspective view. We briefly introduce how to calculate the urban greenness measures based on these views and their relationships with semantic segmentation.

1. Greenness measures based on the plan view

**Figure 3:**
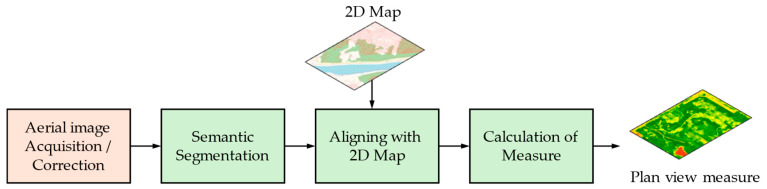
Procedure to calculate a plan view greenness measure.

Figure 3 shows the procedure to obtain a plan view greenness measure. Because aerial images taken by a drone, an airplane, or a satellite inevitably include some form of geometric distortion, it is necessary to correct them for ortho-mapping [41]. There are lots of satellite-imaging programs in both the public and private domains [42]. In addition, the various aerial images (including UAV-captured images) are captured for diverse purposes.

Depending on the type of sensor, aerial images can be categorized into passive and active images. The passive image captures reflected sunlight from objects. On the contrary, the active sensor has its own source of energy and records the reflected information in its image. Usually, LCC, including green-object identification in remote sensing, uses passive images, but active LiDAR data can be solely used or combined with passive images to finely classify the objects, including the types of trees, because the height information can be obtained from the data [43].

Usually, satellite images are characterized by their frequency (spectral), temporal, and spatial resolutions. The frequency (spectral) resolution is the ability of a sensor to discern finer wavelengths (that is, more and narrower bands). Usually, R, G, and B (or with NIR) images are selected for the analysis of the greenness after the postprocessing of multispectral satellite images. The temporal resolution is a measure of the repeat cycles, or frequency, with which a sensor revisits the same parts of the earth’s space [44]. In UGS analysis, sometimes the seasonal effect called Ephemera is important to consider the green-space quality [45]. However, the change in landscape is too slow so that the temporal resolution may not be important.

Spatial resolution (also referred to as the ground-sample distance) refers to the size of one pixel on the ground. For urban greenness, a higher resolution can provide more detailed information to identify objects. For image analysis for greenness measures, very high resolution, smaller than 1 m (e.g., in Maxar’s World View Satellites), has recently been used (refer to Table 1). Satellite imagery is sometimes supplemented with manned or unmanned aerial photography, which has a higher resolution but is more expensive per square meter. The best commercially available spatial resolution for optical satellite imagery is 25 cm [46]. In general, the resolution should satisfy the requirement of the Nyquist rate, which states that the resolution should be at least double that of the fine details that we want to scrutinize [47].

The aerial images after radiometric as well as geometric correction can be classified or segmented to consider the LCC, as explained in Section 3.2. In Figure 3, we just use the semantic segmentation block because patch classification is an old-fashioned method that produces coarser results. For greenness measures, the segmentation of simple thresholding can be applied to each pixel after the feature (band) transform, which results in a binary mask of green objects. The Normalized Difference Vegetation Index (NDVI), Green Ratio Vegetation Index (GRVI), Soil-Adjusted Vegetation Index (SAVI), and Enhanced Vegetation Index (EVI) are examples of the transformed features from the RGB and NIR bands [48]. Similarly, the index to identify water objects can also be defined by the proper feature (band) transform followed by pixel thresholding, which is frequently combined with the greenness to consider the UGS in a wide sense. The accurate DL-based segmentation methods are reviewed in Section 4.

In general, the 2D map of the area of interest (AOI) is aligned with and overlaid onto the segmented results of plan view images to calculate the measure of greenness at the neighborhood scale or city/district scale. The plan view greenness measures calculated in Figure 3 deal with the “available” or “accessible” amount of greenness in terms of the experience of green exposure. When accessibility is considered with an epidemiological study, a buffer zone is usually predefined around residents to calculate the amount of greenness. Sometimes, the road distance instead of the Euclidean distance on a map is considered for the “accessible” greenness measure [7].

2. The perspective view measures

**Figure 4:**
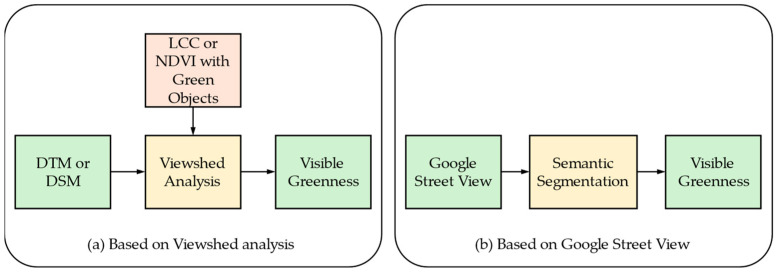
Visible-greenness calculation methods.

Greenness measures based on the perspective view can be calculated by two methods, as shown in Figure 4. In general, these types of greenness are objective and quantitative if we consider merely the amount of visible green objects. However, if we look into the aesthetic view along with green objects, the measure could be qualitative [49]. Also, because the measures take into account the “visibility” of the green exposure, they are frequently calculated based on a residential area or along a road [31,50].

One can use viewshed analysis [51,52] using DTMs (or DSMs) and LCC with green objects, which can be obtained from the plan view images. Viewshed analysis is employed to delineate visible areas from observation point(s) through the geographical calculation of a DTM in the urban area. For instance, if there is a building obstructing the line of sight, the calculation considers the areas behind the building as non-visible. Urban planning researchers assign the locations of green objects once they have data on the visible area [7]. There are various types of green objects that provide data regarding the greenness, such as LCC and the NDVI. Thus, the greenness depends on the fineness of the green-object categories in the LCC or on the spatial resolutions of the LCC. For viewshed analysis, a DTM (DSM) is inevitable to consider the heights. In urban areas where high buildings for residents are densely located, a DTM is desirable, while a DSM is enough when there are fewer man-made obstacles to hide green objects.

There are various types of viewshed analyses, such as binary viewshed, cumulative viewshed, and visual-magnitude (VM) analyses. Also, the greenness can be calculated by forward- or reverse-viewshed analysis. In forward-viewshed analysis, a viewshed with green objects is calculated for a fixed viewpoint. On the contrary, in the reverse viewshed, the viewpoints are collected as a viewshed for a fixed green object [14]. Thus, the reverse viewshed is just the exchange of the role of an object and the observer’s viewpoints. The choice between forward and reverse viewshed frequently depends on the computational complexity, which is related to the number of object points or observer points. The forward (reverse) viewshed is advantageous when the number of viewpoints (objects) is less than the number of objects (viewpoints). The binary viewshed represents a binary mask within which the objects are visible. The cumulative viewshed considers the counts of the number of viewpoints from which it is visible. In the binary or cumulative viewshed, how well the objects are visible is not considered. In VM analysis, the distance between an observer and an object and the viewing angle of an object from the viewpoints are considered to analyze the viewshed.

**Figure 5:**
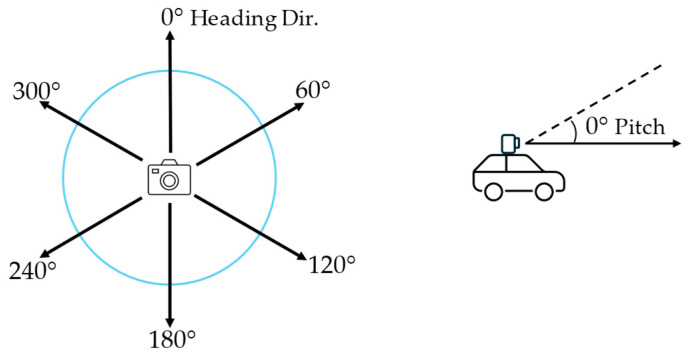
Six directions and pitch to calculate GVI from GSV.

Another direct calculation of visual greenness is the Green View Index (GVI), which has been adapted in many studies [53,54,55,56]. The GVI calculates the greenness exposures from the viewpoint of a car-mounted camera on the street viewing the vegetation in the horizontal direction or viewing the canopy in an elevated direction [16,21]. The GVI measures the visibility of the surrounding greenery at the site of a geographical point on the road. The GVI is based on the images extracted from Google Street View (GSV) for each site, where the images are retrieved from Google Street View API. Usually, six images with 60° intervals are captured for all the surrounding scenery of a site (heading), because four images with 90° intervals could fail to capture objects at 45° directions. The vertical-view angle (pitch) is usually fixed to 0° or parallel to the horizontal line, as shown in Figure 5.

Based on the extracted images, the Green View Index (GVI) [16] is calculated as follows:

(1) $$\mathrm{Green}\;\mathrm{View}\;\mathrm{Index}=\frac{\sum_{i=1}^{n}{Area}_{g_{i}}}{\sum_{i=1}^{n}{Area}_{t_{i}}}$$

where *n* is the number of images for each site, set to six in this study; ${area}_{g_{i}}$ is the number of green pixels in the image for the *i*-th direction; and ${area}_{t_{i}}$ is the number of total pixels in the image for the *i*-th direction.

There are several methods to calculate the ${area}_{g_{i}}$ in Equation (1). One method is to use feature (usually RGB channels) transform followed by thresholding, as in the previous plan view measure. For example [16],

If (Green > Red) and (Green > Blue), then it is a green pixel.(2)

The rationale behind the rule is that green vegetation has high reflection in the green band and low reflection in the red and blue bands. Frequently, the GVI is expanded to the area level (e.g., the block, census tract, or administrative-boundary level) by proper aggregation [57]. Another method uses the semantic segmentation result after image analysis, in which only the areas with green objects are counted. Once the segmentation result is accurate, the addition of the areas is obvious. In the unsupervised segmentation in Treepedia 1 [54], the mean-shift algorithm [58], one of the unsupervised clustering algorithms, was adopted, in which the RGB features are combined with spatial features to obtain spatially lumped segments of objects. Usually, the tree canopy is the only green object to define the GVI, but it is not necessary to confine the green objects to the tree canopy. In this method, semantic segmentation plays an important role for calculating the GVI, and it is one of the major concerns in this review.

Another way in Treepedia 2.0 [17], instead of Equation (1), exploits the DL-based regression method to directly calculate the GVI from an image without semantic segmentation. Here, the authors show that the green objects were correctly identified in the ResNet-50 model [59] by the GradCAM algorithm [60], which is an explainability algorithm that overlays heatmaps onto the areas of input images that primarily influence the decision of a black-box model. The result can be more accurate than the one based on unsupervised or supervised DL-based semantic segmentation.

The simple feature transform-based method in Equation (2) neglects the fact that the pixels in a segment have spatial proximity so that there might be many fragmented green objects after segmentation. In the unsupervised clustering approach, it is possible to misclassify a green painted wall or a shop sign as a green object because the pixel-based RGB feature does not well consider the context information distributed in street images. Although the DL-based regression method in Treepedia 2.0 could produce the accurate greenness measure, it is not possible to extend the greenness to, for example, the quality-included greenness based on the surrounding objects. This is the reason why it is necessary to access the segments of the individual objects to define a more sophisticated greenness measure based on street views.

### 3.2. DL-Based Semantic Segmentation Techniques

**Figure 6:**
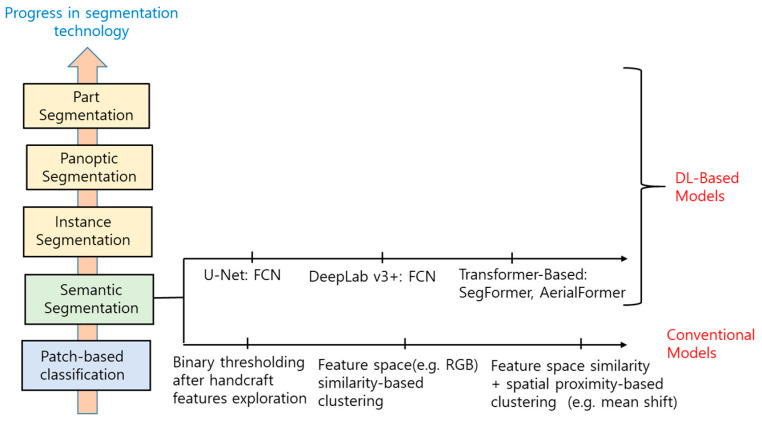
Progress in segmentation techniques mentioned in this section.

There has been significant progress in segmentation technology since DL was introduced. Figure 6 shows a diagram that represents the progress, including semantic segmentation. The figure can be referred to for the identification of the position of a segmentation model that appears in this section. The common technology, regardless of whether the plan view or the perspective view measure of the UGS is used, is semantic segmentation. Because there has been significant progress in DL-based techniques, this section introduces them for the plan view- and the perspective view-based greenness measures. First, the general knowledge on segmentation related to landscape analysis and design is reviewed, and then the DL-based semantic segmentation techniques for the plan view and the perspective view with the metrics for measuring the performance and their public datasets are introduced. Although there have been lots of DL-based semantic segmentation models, we only introduce several representative ones due to the limited number of pages. However, we tried to organize them to allow readers to understand the technological progress in this area.

#### 3.2.1. General Knowledge on Image Segmentation Related to Landscape Analysis and Design

Image segmentation, especially the semantic segmentation called the dense predictor, classifies pixels as spatially connected groups depending on the semantics. In the previous patch-based classification in LCC, the category label is given to a patch (also known as a superpixel), a set of pixels in a rectangle [61], while semantic segmentation tries to classify every pixel. The patch is a set of neighboring pixels (i.e., 4 × 4 or 8 × 8) that can be classified into one of several predefined classes so that it provides a coarser result than the pixel classification called semantic segmentation.

In urban greenness measurement, the green objects in the plan view RGB (or NIR or LiDAR) aerial image are initially classified into trees, shrubs, grass, and other vegetation. Also, in GVI calculation, the green objects, usually the tree covers in a street-view image, are grouped to calculate the GVI. The granularity of the green or related objects in both greenness measures usually depends on the purposes. Sometimes, the coarse categorization of just “Tree canopy” is enough, but the finer categorization would be better for further analysis and planning.

1. From Simple-Binary-Based to Clustering-Based Semantic Segmentation

Usually, the semantic segmentation is performed by two principles: the similarity in the feature space and the spatial proximity [62]. The former tells whether the pixels in a group have similar features, while, in the latter, the pixels in a group should be spatially close to each other to make them lumped. Note that the NDVI in the previous section is one of the transformed features used to find the vegetation areas based on the RGB and NIR bands. Thus, the NDVI followed by thresholding just uses the similarity in the feature space so that there might be a lot of fragmented green areas without postprocessing. Also, the green objects decided by rule (2) exploit only the similarity in the feature space, so that the fragmented segments are inevitable. One can obtain a similar result even if a complicated clustering algorithm based on only feature-space similarity is adopted for the segmentation. This is why unsupervised clustering, such as that based on the mean-shift algorithm [58] to group the green pixels in the spatial domain, was adapted for GVI calculations [16]. Note that there are only two objects to be discriminated against in the examples by thresholding: vegetation (green) objects or non-vegetation objects, although clustering algorithms can have several objects;

2. Recent Segmentation Techniques for Landscape Analysis

**Figure 7:**
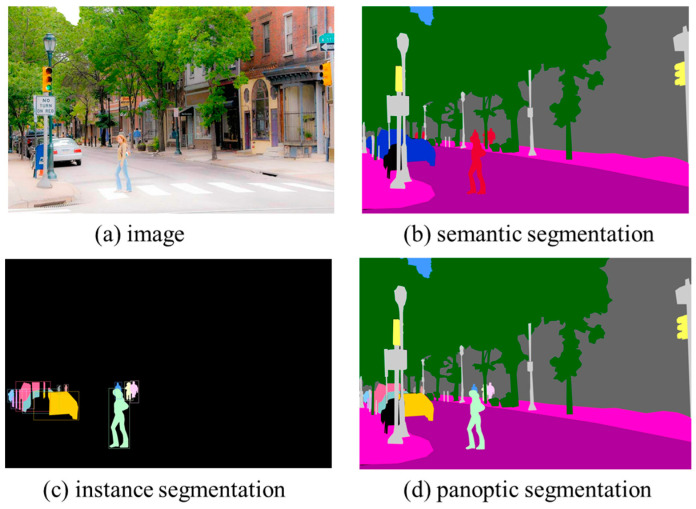
Semantic, instance, and panoptic segmentation in which cars and persons are things and others are stuff (redrawn from Kirillov et al. [64]).

Although the semantic segmentation of green objects is enough for calculating the plan view greenness and GVI, recent image analysis divides the individual instances into a set of semantically meaningful objects by the instance segmentation technique. In the instance segmentation, the “things” that are focused on and that have different instances can be individually identified. Thus, the instances, even in the same categorized objects, are individually localized by bounding boxes to delineate them, and the corresponding labels are given to the boxes. One step further, we can use the panoptic segmentation in which the classes in an image can be grouped into “things” and “stuff”, in which instance segmentation is performed on “things”, while the “stuff” is semantically segmented. Thus, in the results of panoptic segmentation, the same objects belonging to “stuff” have their own color, but the instances in the “things” are individually separated with different colors. Figure 7 shows examples of semantic, instance, and panoptic segmentation. In Figure 7c, the “car” and “person” objects are the “things”, and the others are grouped into the “stuff” category (colored black), while, in Figure 7d, the stuff is semantically segmented into “trees”, “buildings”, and “sky”. In the greenness measurement, green objects, including trees, shrubs, grass, and related objects, could be treated as “things”, and the others, including “sky”, “car”, “road”, “sidewalk”, “building”, etc., could be stuff. The most recent technology tries to divide the “things” into “parts” and to recognize them separately (i.e., the crown and trunk as parts of a tree) [63].

Thus, we can say that the recent DL-based segmentation technology could provide a new horizon in landscape analysis by elaborate segmentation techniques. Researchers could have more chances via finely categorized and individually identified objects than via coarsely or inexactly segmented ones. As well as the segmentation techniques, the higher resolution can produce better-looking small objects, so that the detailed greenness measure, and even the quality of the green space based on the fine semantic granularity, could be defined from the segmentation results.

#### 3.2.2. Deep Learning-Based Semantic Segmentation Models

There are two different deep learning components applied in computer vision that includes segmentation: one is the Convolutional Neural Network (CNN) and the other is the Visual Image Transformer (ViT). These segmentation models have recently been shared via the Python code on GitHub (the model named “mmsegmentation”). To capture image features, the CNN model learns the set of convolution kernels, which is composed of the weighted sum of neighboring pixels. On the contrary, the transformer model refers to the block of pixels by the self-attention operation regardless of the neighboring blocks. There is another important component that appears in the review called the Feed Forward Network (FFN) or Multilayer Perceptron (MLP) structure, which is frequently used for deep learning architectures to transform a feature space into another feature space or decision space. Thus, almost all the DL architectures are basically constructed from these three components followed by nonlinear activation functions in different configurations with subsidiary operational components [65,66,67,68,69].

1. Fully Convolutional Network (FCN)

**Figure 8:**
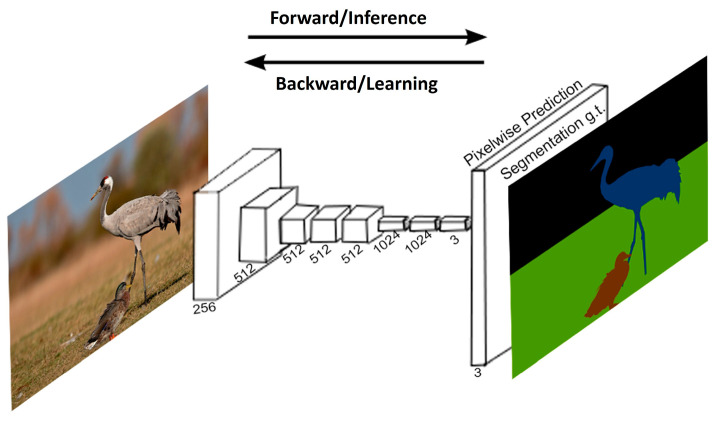
Fully Convolutional Network, emphasizing the encoder part (redrawn from Long et al. [70]).

The major architecture for semantic segmentation based on the CNN is the Fully Convolutional Network (FCN), which automatically transforms an input image (i.e., represented in the RGB space) with other bands to meaningful features represented in feature maps after training a CNN-based encoder structure and categorizes the class of every pixel via a decoder structure. Usually, the encoder structure produces a reduced size but extends the dimension of feature maps (number of learned features) by extracting various meaningful features from an input image. On the contrary, the decoder part consists of the expansion operation to restore the size of the original image by so-called deconvolution or interpolation, followed by the pixel-wise classifier for realizing the semantic segmentation. In general, the FCN consists of CNNs of the encoder-and-decoder structure followed by the FFN for pixel-wise class decisions. A typical FCN structure explaining the simplified overall structure is represented in Figure 8 [70].

Usually, deep learning-based methods can automatically extract meaningful features differently from traditional computer vision, in which domain experts explore and exploit them. The automatic feature extraction is achieved from the learned kernels of the CNN, called the backbone, that usually result from training with substantial amounts of data [71]. In Figure 8, the directional arrows symbolize the alternating forward and backward processes during the learning (training) phase. These processes work to minimize predefined decision losses, and the forward process is executed during the inference stage for semantic segmentation after the completion of training.

In the figure, the feature extraction part from the input image to obtain feature maps is performed in an encoder, while the deconvolution or interpolation for the up-sampling and classification is performed in a decoder. Without the decoder, the structure is the same as that of an image classifier. Because the encoder part plays the same role as the feature extraction in image classifiers, lots of structures pretrained for the classifiers can be adopted to connect with the decoder efficiently to extend and exploit the reduced-size feature maps for pixel-wise classification [72]. In transfer learning, the pretrained encoder after training with a large number of public datasets can be borrowed, and then fine-tuning follows for the specific sematic segmentation with a limited amount of data on purpose. There are two representative FCN structures for semantic segmentation: U-NET and the DeepLab series.

- U-NET

**Figure 9:**
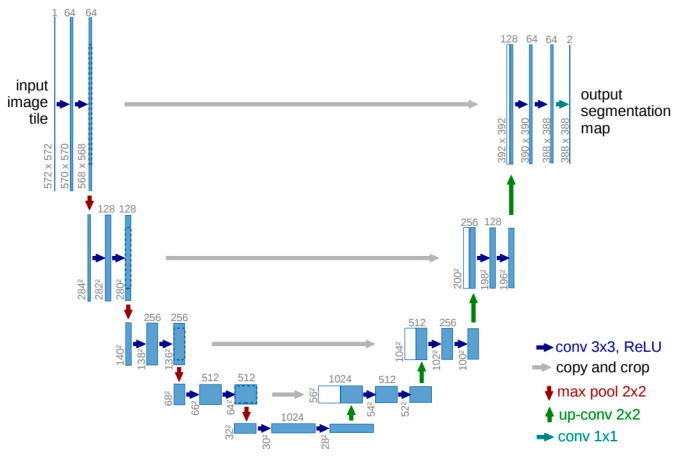
U-Net structure: dividing in half, the left part shows the encoder and the right part shows the decoder (adapted with permission from Ronneberger et al. [73]).

The original U-Net structure is shown in Figure 9 [73]. Dividing the figure into halves, the left half and the right half represent the encoder and decoder, respectively. The consecutive 3 × 3 convolutions with subsidiary operations in the downward direction of the encoder capture the more and more sophisticated high-level features. The 1 × 1 convolution for the output segmentation map is the same as the FFN operation to decide pixel-wise classes. In the figure, there are only two classes assumed: the background and the objects. If there are many objects, then the number of output segmentation maps increases with the increase in the number of classes with different 1 × 1 convolutions. The downward arrow represents the max pooling, which takes the maximum value as a representative among the 2 × 2 pixels. The upward arrow represents the expansion or interpolation, which just copies the same value as for the 2 × 2 pixels. The important characteristic of U-Net is that the decoder part uses both expanded feature maps and the copied ones from the encoder via skip connection for taking convolutions. This can well preserve the low-level features in the decoder part.

In the LCC, the LiDAR data were efficiently combined with RGB aerial images using the U-Net structure, as mentioned in Section 3 of [12], and the Google satellite images were successfully segmented by U-Net [74]. However, the original U-Net is a pretty old model, announced in 2015, so it may be hard to find recent applications. Instead, there are so many variants that improve the performance by changing the backbones of Convolutional Neural Networks (CNNs) [75] or by adding advanced subsidiary components, such as the attention mechanism in Attention U-Net [76]. In all the variants, even in the variant that tries to adapt the transformer, they preserve the shape of the original U-Net, as in Figure 9;

- Google’s DeepLab series

**Figure 10:**
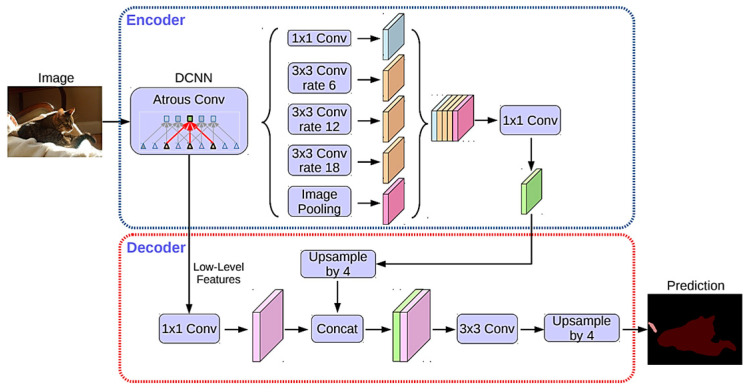
Structure of DeepLab v3+ (adapted from Chen et al. [77]).

The most famous FCN model is the DeepLab series, and DeepLab v3+ is currently the most recent version, shown in Figure 10 [77]. The structural difference in the series from U-Net is in the limited number of interconnections from the encoder to the decoder. Also, the deep convolution layers in the encoder are replaced with different rates of atrous (dilated) convolutions, called the Atrous Spatial Pyramid Pooling (ASSP) module. The atrous convolution with a larger dilation rate can effectively increase the receptive field that implies the range of the visual field from which the contextual features are extracted, without increasing the CNN layers [78]. There are diverse dilation rates with fixed sizes of convolution in the encoder to well capture multi-scale image features. In the decoder, the low-level features after expansion are combined with up-sampled multi-scale features to make pixel-wise class decisions after magnification.

2. Visual Transformer (ViT) and ViT-Based Segmentation

In the context of natural language processing (NLP), recurrent and CNN models based on encoder-and-decoder architectures were commonly applied before the emergence of the transformer models. However, the transformer eliminates the recurrent and convolution layers and proposes a simple model based entirely on the self-attention mechanism, in which each word (token) attends to every other word in the same input sequence. As a result, the transformer model takes significantly less time to train than its counterparts, and with a better performance while achieving more parallelization [79]. In computer vision, the tokens in NLP are replaced by the patches of an image, and then similar self-attention among the patches is carried out. After successfully announcing ViT-based classifiers, various computer vision tasks were solved by the transformer-based structure, including semantic segmentation. The fundamental difference between the operations in transformers and CNNs is in the range of operations in self-attention and convolution. Transformers efficiently handle dependencies across larger distances with the self-attention mechanism, while CNNs try to capture local features by neighboring convolution operations. Because the evolution of DL-based segmentation techniques is so fast, this review introduces only two recent models based on two different structures.

- Basic ViT Classifier Model

**Figure 11:**
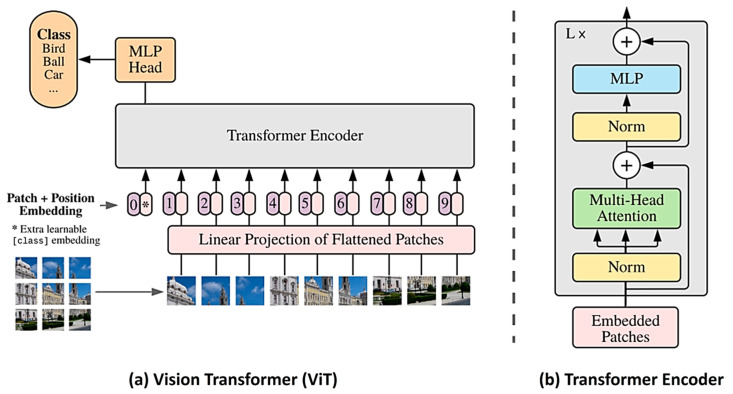
Structure of the ViT for classification (**a**) and internal structure of the transformer encoder (**b**) (adapted with permission from Dosovitskiy et al. [80]).

In computer vision, Dosovitskiy et al. first applied a pure transformer block on a sequence of image patches, termed the Visual Transformer (ViT) [80]. According to the study, the input image is split into fixed-sized patches, each with 16 × 16 pixels, and treated in the same way as a single token is treated in NLP. The patches are then flattened and undergo trainable linear projection. Positional embedding vectors are added to each input patch and the class token is prepended and then feed-forwarded through a transformer encoder, which consists of L consecutive blocks based on multi-head self-attention (MSA). In each block in the encoder, there is an MSA module followed by an MLP module. A LayerNorm (LN), a kind of subsidiary operational component, is applied before the MSA and MLP modules, and a residual connection is also applied before the LN layers, as shown in Figure 11. After propagating the tokens through the encoder, the randomly initialized class token can accumulate information from the other tokens in the token sequence the deeper and more layers that the transformer goes through. An MLP-based head that only refers to the information at the last layer’s class token is used for the class decision;

- Swin Transformer

**Figure 12:**
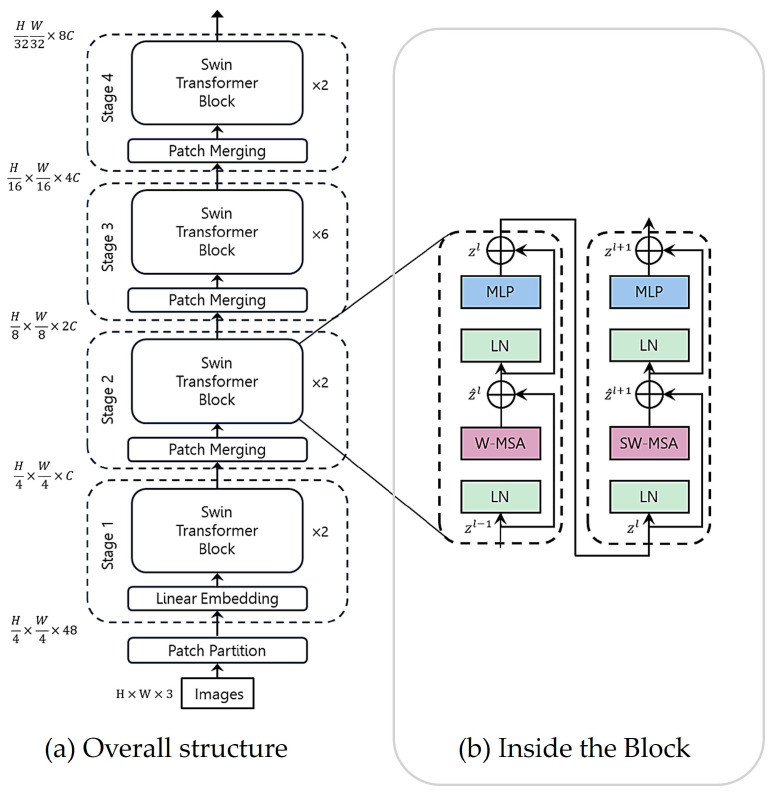
Structure diagram of Swin Transformer: overall architecture (redrawn from Liu et al. [81]).

The Swin Transformer replaces the standard block of the ViT in Figure 11b with the Swin Transformer block shown in Figure 12b [81]. In Figure 12b, the Swin Transformer block consists of pair-shifted, window-based Multi-Head Self-Attention (MSA) modules, each of which is followed by a two-layer MLP module. An LN is applied before each MSA and MLP module, and a residual connection is also applied before each LN operation. In addition, the Swin Transformer also uses the hierarchical feature map constructed by the Patch Merging block to compute the representation of the input, which is similar to the downsizing of the spatial dimension in the U-Net encoder. In Figure 12a, the Patch Merging cuts the number of tokens in half in each dimension so that the number of tokens is reduced from *H*/4 × *W*/4 to *H*/32 × *W*/32, while the Swin Transformer block doubles the number of channels from *C* to 8*C* as the stage goes on.

One of the advantages of the Swin Transformer compared to the ViT is its smaller token size, which implies that the fine detail of the local features of an image can be well represented. The patch size is 4 × 4 in the Swin Transformer, while it is 16 × 16 in the ViT. However, this could also increase the computational complexity in the MSA operation because a patch could refer to too many patches in an image. However, the Swin Transformer confines the reference within the fixed size of 7 × 7 windows so as to reduce the range. Instead, to compensate for the limited range of the references in the window-based W-MSA operation in the first block, the second-shifted window-based SW-MSA operation is performed in the second block, as in Figure 12b. The pair of MSA blocks efficiently overcomes the locality of the attention operation while reducing the computational complexity.

Figure 12 shows only the encoder part of the Visual Transformer to extract image features. The structure of the Swin Transformer has been adopted in many structures of computer vision tasks, including semantic segmentation [82]. Also, Swin U-Net uses the Swin Transformer and its reverse operations for the encoder and decoder structures, respectively, in the U-Net structure for semantic segmentation [83]. Later, AerialFormer adopted the same concept as Swin U-Net in the encoder for the semantic segmentation of aerial images;

- SegFormer for Semantic Segmentation

**Figure 13:**
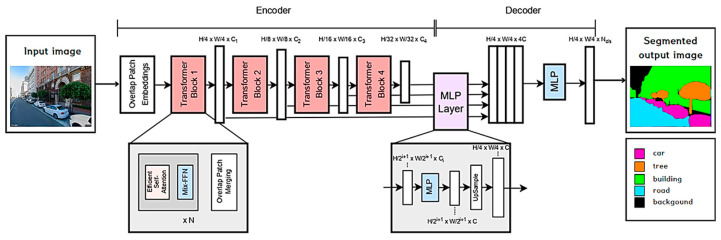
Structure of SegFormer for semantic segmentation (redrawn from Libo et al. [84]).

SegFormer is a recent semantic segmentation model based on the Visual Transformer [84]. Figure 13 shows the basic structure of SegFormer, which consists of two sections: the encoder and the decoder. The encoder outputs multi-scale features, and the decoder aggregates this multi-scale information from different layers with MLPs to perform semantic segmentation. For a better performance in pixel-wise class prediction, the input image to the encoder is first divided into 4 × 4 patches, like in the Swin Transformer [81]. Each transformer block in the encoder is composed of three sub-modules: Efficient Self-Attention, the Mix-Feedforward Network based on the FFN, and Overlapping Patch Merging. Efficient Self-Attention is similar to the Multi-Head Self-Attention in the original ViT model, which helps to lower the computational cost of the self-attention process. Differently from the ViT, however, SegFormer does not have the fixed-resolution Position Encodings (PEs). Instead, it inserts a 3 × 3 convolution into the FFN to incorporate data-driven positional information. Lastly, the Overlap Patch Merging block is used to reduce the feature map size and preserve the local continuity, as in the Swin Transformer. This results in hierarchical feature representation of 1/4, 1/8, 1/16, and 1/32 sizes of the original image resolution.

The decoder modules contain a full-MLP layer, which takes the features from the encoder module and aggregates them together. The process is performed in four steps. First, multi-level features from the encoder go through an MLP layer for unification in the channel dimension (C). The features are then up-sampled to 1/4 of the original sizes, *H*/4 × *W*/4, and concatenated together to produce 4C feature maps. An MLP layer then concatenates the up-sampled features to make C feature maps. Finally, an MLP takes these fused feature maps to predict the final segmentation mask of a *H*/4 × *W*/4 × $N_{cls}$-sized resolution, where $N_{cls}$ stands for the number of categories;

- AerialFormer for semantic segmentation of aerial image

**Figure 14:**
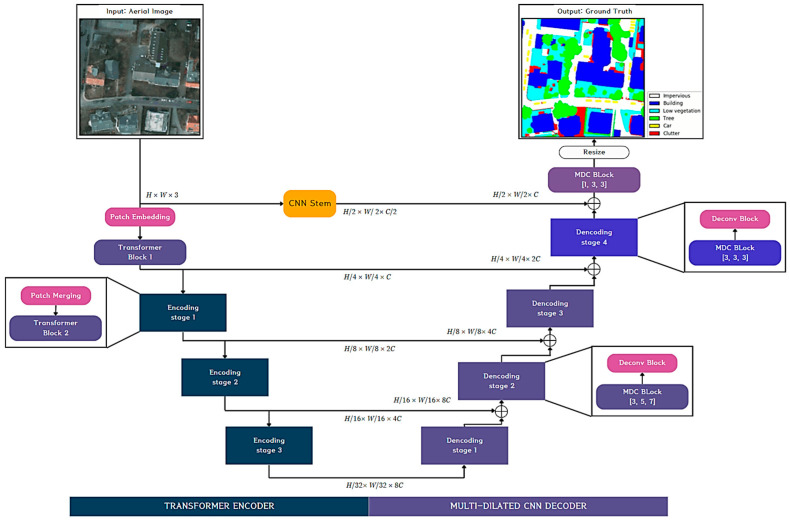
Structure of AerialFormer (adapted and redrawn from Yamazaki et al. [85]).

Because the plan view greenness measure assumes LCC or the semantic segmentation of green or related objects, we introduce AerialFormer, a recently developed Visual Transformer model that is specialized and records a state-of-the-art performance for the semantic segmentation of aerial images [85]. There are several challenging characteristics in aerial image analysis, such as the strong imbalance in the foreground (things)–background (stuff) distribution, the complex background, intraclass heterogeneity, interclass homogeneity, and tiny objects. The authors argue that their model can handle these problems by unifying the transformers at the contracting path with lightweight Multi-Dilated Convolutional Neural Networks (MD-CNNs: MDC) at the expanding path, as shown in Figure 13. Note that the overall structure resembles U-Net, but the encoder adopts the Swin Transformer in each stage. The decoder consists of multi-stages of an MDC block followed by a deconvolution to expand the dimension. The MDC block is defined by three parameters [r1, r2, r3] corresponding to three receptive fields, and it consists of three parts: the Pre-Channel Mixer, Dilated Convolutional Layer (DCL), and Post-Channel Mixer. In the MDC block, DCL plays a similar role to ASSP in DeepLab v3+, and the Pre-Channel Mixer and Post-Channel Mixer mix the features and change the dimension before and after the DCL. Also, the CNN stem takes part in preserving the low-level features in the same way as in DeepLab v3+. It is worthy to note that the decoder part in SegFormer in Figure 12 is changed into a more elaborate structure in Figure 14, which was adapted from U-Net to improve the performance.

#### 3.2.3. Performance Metrics for Semantic Segmentation

The performances of the semantic segmentation models are validated and compared to each other with public datasets and with various metrics [86]. The most popular metrics are the mean intersection over union (mIoU) and pixel accuracy (PA). For the calculation of the mIoU, the IoU is defined as follows:

(3) $$IoU=\frac{predicted\;mask⋂ground\;truth\;mask}{predicted\;mask\;⋃ground\;truth\;mask}$$

where the predicted mask results from the model, and the ground-truth mask is given by labeled data by polygons from a human expert. If the IoU approaches 1 (0), then the model produces pretty close (far) to (from) the ground-truth mask. Usually, there are several objects, including things and stuff to be segmented. Thus, the mIoU is calculated by taking the average of all the IoUs of the objects. Note that the boundaries of natural objects, such as trees and shrubs, are harder to capture by polygons compared to man-made objects, including buildings, roads, etc. Thus, it is almost impossible for natural objects to obtain an IoU close to 1.

The PA is calculated as follows:

(4) $$PA=\frac{\sum_{i=1}^{K}p_{ii}}{\sum_{j=1}^{K}\sum_{i=1}^{K}p_{ij}}$$

In the above equation, $p_{ij}$ stands for the number of pixels in the predicted class (j), the true class is which is i. Thus, the denominator implies the size of the image (i.e., the whole number of pixels), and the numerator is the number of correctly classified pixels when there are *K* object classes. Then, the mean PA (mPA) of all the test images can be calculated.

In Treepedia 2.0, only the single object of tree cover is considered in the calculation of the IoU. Also, it introduces the Mean Absolute Error (MAE) for the performance measure of the GVI. In order to calculate the MAE, the number of misclassified pixels for the tree-cover object is counted first, and then an average is taken over all the test images. Thus, the MAE is the same as the average of (1-PA) in Equation (4), in which only a single object is considered [17].

#### 3.2.4. Related Datasets for Greenness Measures

In UGS analysis, the public datasets can be used for two purposes in the same ways as in other areas: (1) to evaluate the DL models and compare them with each other, and (2) to perform transfer learning to overcome the lack of labeled data. For the evaluation and comparison purposes, it is obvious that the dataset for training and evaluating DL models should the same. In general, however, it is not easy to build a large number of annotated data in a short time period, even with crowdsourcing. So, in transfer learning, the public dataset of a similar property to the task at hand can be chosen for pretraining a DL model, and then the pretrained model can be fine-tuned with a smaller number of self-built datasets.

There are various open-source public datasets and semantic segmentation models [87]. Although they are not directly related to UGS analysis with greenness measures, some are indirectly related. One could choose a proper public dataset that has green objects (with related ones) among the “things” in the dataset. The datasets are categorized into two types: one for the plan view and another for the perspective view greenness measures. Note that new datasets for various domains including UGSs are continuously constructed and announced. This is why the datasets mentioned in this review are only a part of them.

1. Datasets for the Plan View Greenness Measure

Datasets for LCC related to the plan view greenness are listed in Table 1. Some of them have classes labeled vegetation and forest for urban as well as rural areas. Here, we review several datasets in more detail. It is worthy to note that the datasets in Table 1 are not constructed exclusively for measuring the plan view greenness. For fine-grained greenness measures for landscape analysis, it is necessary to construct a high-resolution dataset that includes lots of landscape components as the categories to be segmented.

**Table 1:** The Plan view-based image dataset (adapted from Wang et al. [88]).

Image Level	Resolution (m)	Dataset	Year	Sensor	Class	Image Width	Images
Meter level	10	LandCoverNet	2020	Sentinel-2	7	256	1980
	4	GID	2020	GF-2	5	4800~6300	150
Sub-meter level	0.6	Zurich Summer	2015	QuickBird	8	622~1830	20
	0.5	DeepGlobe	2018	WorldView-2	7	2448	1146
	0.25 (0.5)	LandCover.ai	2020	Airborne	3	4200 (9500)	41
	0.05	Zeebruges	2018	Airborne	8	10,000	7
	0.05	ISPRS Potsdam	2013	Airborne	6	6000	38
	0.09	ISPRS Vaihingen	2013	Airborne	6	1887~3816	33
	0.3	LoveDA	2021	Spaceborne	7	1024	5987

- ISPRS Potsdam and Vaihingen datasets

**Figure 15:**
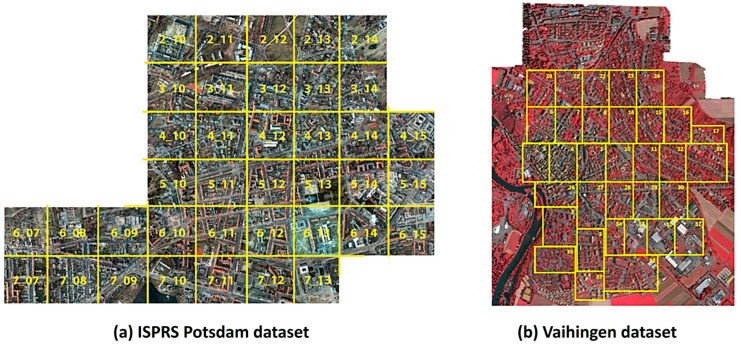
ISPRS Potsdam dataset (**a**) and Vaihingen dataset (**b**), adapted from ISPRS [88,89].

The Potsdam dataset [88] contains 38 high-resolution (5 cm) images of 6000 × 6000 pixels over Potsdam City, Germany. There are two modalities included in the Potsdam dataset: true orthophotos (TOPs) and digital surface models (DSMs). For the plan view greenness, one can choose TOPs, which correspond to RGB images. There are six categories of objects: impervious surface, building, tree, low vegetation, car, and background, among which the tree and low-vegetation categories are related to urban greenness. The NIR band is also available to combine with RGB images. Similarly, the Vaihingen dataset [89] contains 33 high-resolution images (of different sizes) with a resolution of 9 cm. It also includes both true orthophotos (TOPs) and digital surface models (DSMs). The categories of objects are similar to those of the Potsdam dataset. Figure 15 shows the Potsdam (left) and Vaihingen (right) datasets.

The ISPRS Potsdam dataset was exploited to develop the U-Net-based multitasking technique of semantic segmentation with the Cityscape dataset, which is a perspective-view dataset. In multitasking semantic segmentation, a pair of heterogeneous plan view and perspective view data points are simultaneously applied to U-Net with two heads to produce the semantic segmentation results [90]. ISPRS Vaihingen was used to obtain the experimental results to show the validity of Stacked Fully Convolutional Networks (SFCNs), in which various DL-based sematic segmentation models are parallelized and aligned to obtain the aggregated result [91];

- LoveDA and LandCover.ai datasets

The LoveDA dataset consists of 5987 high-resolution images of 1024 × 1024 pixels with a 30 cm spatial resolution. The data include 18 complex urban and rural scenes and 166,768 annotated objects from three different cities (Nanjing, Changzhou, and Wuhan) in China. The number of classes is seven: background, building, road, water, barren, forest, and agriculture. The LoveDA dataset encompasses two domains (urban and rural), which brings considerable challenges due to the (1) multi-scale objects; (2) complex background samples; and (3) inconsistent class distributions. Thus, the LoveDA dataset is suitable for both land-cover semantic segmentation and unsupervised domain adaptation (UDA) tasks (e.g., from urban (rural) to rural (urban)). Later in the review, UDA is discussed in more detail. Wang et al. compared the performances of diverse semantic segmentation models and UDA methods [92].

The LandCover.ai dataset was built for the automatic mapping of buildings, woodlands, water, and roads from aerial images. It took images from Poland, Central Europe, with around 9000 × 9500 (4200 × 4700) pixels of RGB spectral bands, which consisted of 33 (8) orthophotos with a 25 (50) cm per pixel resolution [93];

- Very-High-Resolution (VHR) Zurich Summer v1.0 and IEEE Zeebrugge (grss_dfc_2015) datasets

The Zurich Summer v1.0 dataset is a collection of 20 chips (crops), taken from the QuickBird acquisition of images of Zurich, Switzerland. QuickBird images are composed of NIR and RGB bands and were pansharpened to the PAN resolution of 6.2 cm. The labeled objects in the images include roads, buildings, trees, grass, bare soil, water, railways, and swimming pools. The VHR Zurich Summer dataset was employed for semi-automatic semantic segmentation using a DL approach, specifically with a method known as SideInfNet [94]. This technique incorporated brush annotations, enhancing the dataset’s semantic segmentation capabilities.

The IEEE Zeebrugge dataset was provided from the 2015 IEEE GRSS Data Fusion Contest. It was acquired via an airborne platform over the urban and harbor areas of Zeebrugge, Belgium. The whole dataset consists of seven orthophoto images with a size of 10,000 × 10,000, five of which have ground truth (ID Tiles 1, 2, 3, 5, 7), and the remaining two images (ID Tiles 4 and 6) are undisclosed by the organizers for testing. These tiles are eight-bit TIFF files with the R, G, and B bands, with a resolution of 5 cm. The ground-object classes and the corresponding ground-truth colors in the dataset are as follows: impervious surface: white; buildings: blue; low vegetation: cyan; trees: green; cars: yellow; clutter: red; boats: pink; and water: dark blue. Deng et al. [95] proposed a new transformer-based VHR aerial semantic segmentation model named Crisscross Global Vision Transformers. The model consists of two parts: a crisscross transformer encoder block (CC-TEB) and a global-squeeze transformer encoder block (GS-TEB), which increase the local and global feature representation abilities, respectively. The model was validated using the IEEE Zeebrugge dataset along with the LoveDA and ISPRS Vaihingen datasets. Also, Sun et al. [13] suggested a DL-based fusion model in which multimodal VHR aerial images and LiDAR data and the corresponding intramodal features are simultaneously and adaptively combined for better semantic segmentation. In the experiment, the authors used the IEEE Zeebrugge dataset as well as the ISPRS Potsdam and Vaihingen datasets.

2. Datasets for GVI

There are several public datasets that can be used for semantic segmentation to construct the GVI. Here, we introduce two well-known datasets: the Cityscape dataset and the ADE 20K dataset. It can be emphasized that the following datasets are not constructed exclusively for measuring the GVI or for landscape analysis. For fine-grained greenness measures for landscape analysis, it is necessary to construct a dataset that includes lots of landscape elements as the categories to be segmented.

- Cityscape Dataset [96]

**Figure 16:**
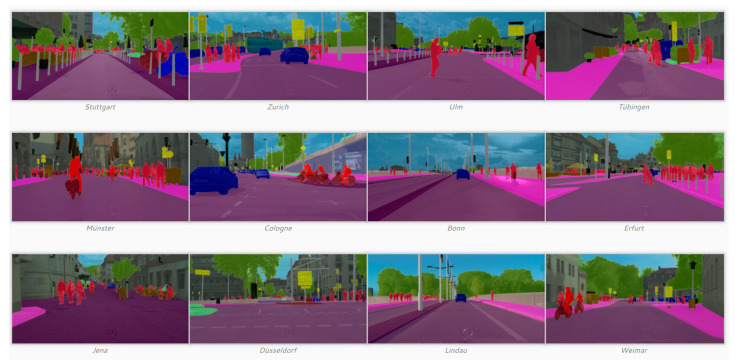
Example of Cityscape dataset (adapted from Cityscape dataset website [96]).

The Cityscapes dataset focuses on the semantic understanding of urban street scenes. It is very good for accurately measuring the GVI because the conditions for image capturing are the same. This dataset has 30 classes, including the landscape components along roads, such as vegetation. Also, the dataset is quite diverse because the images were captured from 50 different cities and during three different seasons: spring, summer, and fall. The amount of data is quite enough, even with 5000 fine annotated images. One could divide the vegetation or terrain classes into several green subclasses, including shrubs, trees, and other green objects, and try to obtain fine-grained green subclasses. Figure 16 shows an annotated image in the dataset. In Treepedia 2.0, the Cityscape developers used the vegetation class to pretrain the ResNet-Based GVI regression model and then fine-tuned the model with their own labeled data. Also, J. Zhang et al. [50] exploited the Cityscape dataset for training the HRNet-OCR semantic segmentation model to calculate the GVI, which was used for finding the GVI-best path in Osaka city, Japan;

- ADE20K Dataset [97]

**Figure 17:**
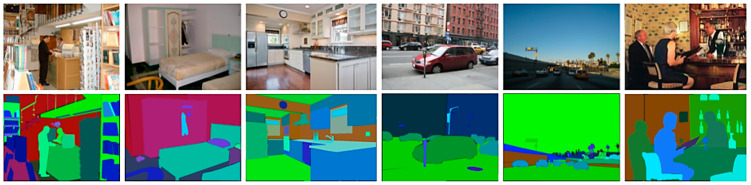
ADE20K semantic segmentation dataset with indoor and outdoor scenes (adapted from ADE20K [97]).

The ADE20K dataset contains about 20 K annotated images of both indoor and outdoor scenes with 150 classes. There are many subclasses related to landscape components, such as rivers, tree, grass, and so on. Thus, this dataset can be used for GVI measurement or landscape quality analysis. The number of images is enough to train and validate for specific landscape applications, including the GVI measure. The MIT Scene Parsing Benchmark (SceneParse150) provides a standard training and evaluation platform for the algorithms of scene parsing, the data of which come from the ADE20K dataset. The segmentation model trained by ADE20K can be used for parsing the green objects or other landscape components for landscape analysis after postprocessing. The ADE20K outdoors3 dataset is a 5000-image subset of the 20,000-image ADE20K dataset. Figure 17 shows example images of the dataset. There are many semantic algorithms in the literature, including ONE-PEACE [98], that exploit the ADE20K dataset.

There are two important things we can point out about the ADE20K dataset: (1) It is a densely annotated dataset that spans diverse annotations of scenes, objects, parts of objects, and, in some cases, even parts of parts. This property enables scene parsing through which a semantic understanding of visual scenes, the holy grail of computer vision, is possible [99]. (2) Therefore, it might be useful to parse the landscape scene with its components for further analysis. Here, we could use instance, panoptic, and part-segmentation techniques for automatic scene parsing.

## 4. Discussion

There could be a lot of issues to be addressed in DL-based segmentation for urban greenness measures based on the plan view and the perspective view. It is obvious that landscape researchers have more choices for analysis and planning due to the technological breakthroughs in DL-based segmentation mentioned in Section 3.2. Among them, the quality of green spaces, including landscape aesthetics, may be one of the areas of research. Moreover, AI application engineers can continuously support the field of planning by suggesting feasible solutions for further research on landscape analysis and urban planning [100].

This section addresses two of them: the application of DL-based segmentation for general landscape analysis and planning, and the unsupervised domain adaptation (UDA) of a DL-based segmentation model with the proper dataset.

### 4.1. DL-Based Segmentation for Landscape Analysis and Planning

Advanced DL-based segmentation is essential for accurate and fine-grained greenness measures. Also, there are lots of potential applications in landscape analysis and planning that require advanced semantic segmentation technology. In this section, we choose the terminology “DL-based segmentation” to include “instance” and “panoptic” segmentation as well as “semantic” segmentation. DL-based “object detection” can be merged into “instance” segmentation; thus, it is not explicitly mentioned in this section.

Of all the applications mentioned in this section, the concept of “explainability” is necessary to understand. Because planning is the reverse process of analysis, the landscape analysis should be explainable for planning. Unfortunately, however, the “explainability” of DL-based segmentation technology is just in the development stage and cannot provide complete reasons for the analysis results. Thus, the statistically well-developed analysis techniques that consist of segmentation followed by explainable regression seem to be inevitable for a while, until the explainable AI is satisfactory for landscape planning.

#### 4.1.1. Toward Fine-Grained Greenness Measures

Segmentation is not the ultimate goal but is an important and necessary intermediate step for measuring the urban greenness and other landscape analyses. As in Treepedia 2.0, semantic segmentation may not be necessary to calculate the GVI. In other words, one can directly obtain an accurate GVI from street images by measuring the relative area of the tree cover by a sigmoid activation function, and the reason for the GVI results can be partially expressed by an explainable AI algorithm, like GradCAM [17].

The application of the segmentation results in this review is focused on the urban greenness measure, in which the “things” of green objects to be segmented are a little restricted. For example, the traditional plan view greenness measure from aerial images is just the NDVI or tree cover in a buffer area, and the GVI considers the single object of tree cover. In general, however, the “things” for greenness measures from aerial images or street-view images should depend on the objects of interest related to the UGS, and they must be application-dependent. In other words, the plan view measures or the GVI could include many fine-grained green objects (e.g., shrubs, grassy areas, or flower gardens) for fine-grained analysis. Incomplete technology has likely been one of the obstacles to considering various or fine-grained “things” for urban greenness. Because the DL-based (semantic) segmentation techniques are rapidly progressing these days, researchers will soon be free from such technological difficulties.

#### 4.1.2. Other Types of Qualitative Landscape Analysis

In addition, it should be emphasized that the “things” of segmentation results can be used in diverse landscape analysis, as well as the greenness measures, once they are successfully segmented. In general, the segmentation results of plan view images can be used in various ways. One can analyze the shape of green spaces, called the greenness morphology [40,101]. Also, it might be interesting to consider a graphical representation of the landscape components after segmentation, including green objects in a garden, for evaluation in terms of the quality of the green space. Graph neural networks (GNNs) [102] could be properly exploited for the representation and evaluation in terms of the diverse qualitative criteria of landscape analysis.

**Figure 18:**
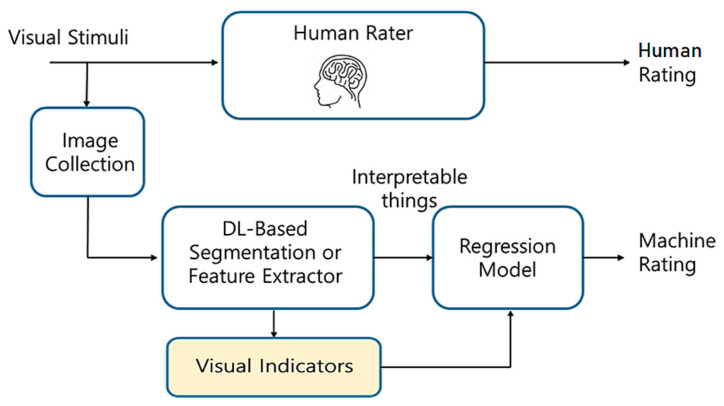
Necessary steps of DL-based segmentation for qualitative evaluation by a machine.

Also, the segmentation results of street-view images can be used in various ways. The initial SBE (Scenic Beauty Estimation) is based on the semantic segmentation of prospectively captured photographs [49]. Furthermore, F. Zhang et al. [100] recently trained DL models to predict human perceptions of street-view images. The model achieved a high accuracy rate in predicting six human perceptual indicators: safe, lively, beautiful, wealthy, depressing, and boring. The results help researchers and urban planners to understand the interactions of place sentiments and semantics. To interpret the rating by the DL model, the researchers obtained 150 object categories segmented from street-view images to calculate the correlations between various objects and each of the six perceptual indicators. Actually, the explainable AI can partially explain the reasons why the DL-based black-box model evaluates the images in terms of qualitative criteria [103]. However, the explainable AI is not complete to help the analysis for planning. This is one of the reasons why DL-based segmentation, as shown in Figure 18, is necessary for the time being.

In addition, there are many other examples that show that segmentation is inevitable for defining indicators for qualitative landscape evaluations. For example, to define the bikeability, exact semantic segmentation is essential to define the proper indicators [104]. Also, there are lots of indicators for visual perception to evaluate landscape aesthetics that can be replaced by or combined with the DL-based model, once enough data for training are available. Unfortunately, the current state of DL technology cannot provide complete and clear explanations, and so semantic segmentation is an inevitable intermediate process to link the visual stimuli to the qualitative indicators for qualitative evaluation [105]. The lower yellow box in Figure 18 represents the necessary path for such qualitative evaluations for landscape analysis and planning.

There is another area of research on the aesthetic evaluation of landscapes that requires the semantic segmentation results of both plan view and perspective view images. The research is just in the beginning stage and requires other information (e.g., DSM). However, plan view image segments could be associated with perspective view ones [106,107] not only to evaluate the SBE but also to relate the evaluation to landscape planning.

### 4.2. Domain Adaptation and Datasets

In Section 3.2.4, we mention the datasets for DL-based semantic segmentation for UGS analysis. However, plan view images obtained by various aerial vehicles especially have their own characteristics because the equipped sensors, the time, and the places for capturing images are different. For example, even the same objects (e.g., a tree species) can have different sizes due to differences in the spatial resolutions and locations, and the surrounding contexts of these objects are diverse. Thus, DL-based segmentation models trained with public datasets are not well suited to the real application of “wild tasks” to analyze the landscape or measure the urban greenness. For this reason, domain adaptation (DA) techniques to reduce the “domain gap” are an important area of research in remote sensing. Among the DA techniques, unsupervised domain adaptation (UDA) is a useful technology because it transfers knowledge learned from the source domain, with a large number of annotated training examples, to target domains with unlabeled data only.

For LCC in remote sensing, there are two types of UDA: one for the classification task [108] and the other for semantic segmentation. In UDA for DL-based semantic segmentation, recent works have mainly proceeded in two directions: self-training and adversarial training. Self-training involves alternately generating pseudo-labels on the target data and fine-tuning the model [109,110].

Although there are many self-training and adversarial training methods for DL-based semantic segmentation techniques that have been recently developed in remote sensing [110], here, we introduce an interesting adversarial approach for measuring the urban greenness of 31 major cities in China [18].

**Figure 19:**
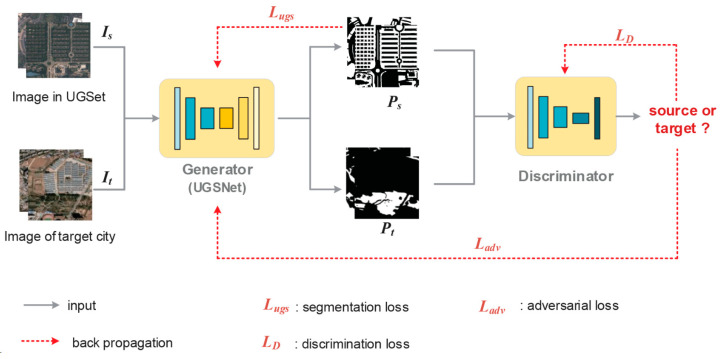
Adversarial approach for urban greenness measurements of 31 major cities in China (adapted from Shi et al. [18] under the terms of a CC BY 4.0 License).

The authors of [19] constructed a large-scale, high-resolution, urban-green-space dataset (UGSet), which contains 4544 images of a 512 × 512 size with a spatial resolution of nearly 1 m. The images in UGSet (source data) were collected from 142 sample areas in Guangdong Province, China, through the Gaofen-2 (GF2) satellite. The authors divided the urban green space into five fine-grained categories: parks, green buffers, square green spaces, attached green spaces, and other green spaces, which is not only diverse but also has large inter- and intraclass-scale differences. Then, they trained the CNN-based semantic segmentation model, the Generator in Figure 19. Again, they prepared Google Earth high-resolution satellite images of 31 major cities (target data) in China with a spatial resolution of nearly 1.1 m. In the adversarial learning with the Discriminator in Figure 19, the target data are segmented as if they are not discernable, whether the results come from source or target data.

This study suggests a couple of research directions to obtain the fine-grained segmentation results of UGS and landscape analysis. As mentioned, successful UDA technology can reduce the efforts to annotate unlabeled target datasets if they are sufficiently close to the source datasets to be domain-adapted. Thus, the recent transformer models that produce an improved performance for semantic segmentation can be successfully applied to UDA for better results without labeling [19,111]. In addition, the DL-based segmentation for specific sets of “things” can be applied to large amounts of unlabeled data for landscape analyses that utilize the same set or subset of labels (things). Note that the set of things for landscape analysis depends on the applications, so that it may be hard to universally specify. Thus, the dataset constructed for an application is hard to use for another application. Thanks to successful UDA, however, one can construct only an appropriate amount of the labeled dataset with the set of fine-grained labels and use a large amount of unlabeled data.

## 5. Conclusions

Rapid, world-wide urbanization and population densification in urban areas can lead citizens to serious physical or mental health problems and eventually result in adverse effects on human well-being. This paper reviews the recent technological breakthroughs in DL-based semantic segmentation, which can provide proper solutions for the requirements of urban greenness measures in landscape analysis. In this review, the measures are categorized into two groups: the plan view-based (ortho-mapped) and the perspective view-based (profile view) images. The plan view-based measures of greenness are the same as those of LCC with green objects. Moreover, the perspective view-based ones can be calculated by viewshed or street-view photograph analysis. This paper covers the GVI based on street-view photograph analysis because the visual greenness from viewshed analysis can be calculated from the plan view measurements with the additional information of DTMs (DSMs).

We started with the background of the urban greenness measures to help with a better understanding of this review. Then, we briefly mentioned the diverse recent segmentation tasks that are potentially available for advanced landscape analysis. The greenness measure is only a specific application for semantic segmentation, in which the objects to be segmented, called “things”, are confined to green objects. In the review, the semantic segmentation techniques spanned from old-fashioned thresholding after handcrafted feature exploration to the recent DL-based CNN and Visual Transformer models that include learned feature extraction. Basically, in an encoder–decoder structure, the encoder extracts the learned multi-scale features, and the decoder combines them to produce pixel-wise classification. The basic operations in a CNN-based model are based on neighboring pixels, while the Visual Transformer uses self-attention. We introduced widely applied CNN-based semantic segmentation models and recent high-performance transformer models. Along with the typical performance metrics of semantic segmentation, this review introduces various public datasets for constructing greenness measures via DL-based semantic segmentation, which requires large numbers of labeled data. Also, the DL-based semantic segmentation models that use public datasets are referenced.

As DL-based segmentation technology progresses, landscape researchers will have better chances to find the answers to their research questions, and AI application engineers will be able to provide feasible solutions to help their research. In the Discussion Section, we point out that accurate (semantic) segmentation is inevitable not only for the accurate and fine-grained greenness measures but also for qualitative landscape analysis due to the incomplete explainability of deep learning-type black-box models. In addition, the unsupervised domain adaptation problem in aerial images is addressed to overcome the issues of scale changes and the lack of labeled data, especially in fine-grained plan view greenness measures. Because the technology for DL-based semantic segmentation is rapidly developing and the pages for the review are limited, we could not deal with all the recent DL-based segmentation techniques. However, the important concepts of the current DL-based segmentation technology are included for further readings. We hope this review can help researchers extend their knowledge on the status of DL-based segmentation technology and obtain the steppingstones for challenging topics in UGS research.

## Data Availability

No new data were created or analyzed in this study. Data sharing is not applicable to this article.
